# Large scale genomic reorganization of topological domains at the *HoxD* locus

**DOI:** 10.1186/s13059-017-1278-z

**Published:** 2017-08-07

**Authors:** Pierre J. Fabre, Marion Leleu, Benjamin H. Mormann, Lucille Lopez-Delisle, Daan Noordermeer, Leonardo Beccari, Denis Duboule

**Affiliations:** 1 School of Life Sciences, Ecole Polytechnique Fédérale, 1015 Lausanne, Switzerland; 2 Department of Genetics and Evolution, University of Geneva, 1211 Geneva 4, Switzerland; 3 Present address: Institute for Integrative Biology of the Cell (I2BC), CEA, CNRS, Université Paris-sud, University Paris-Saclay, 1 Avenue de la terrasse, 91198 Gif-sur-Yvette, France

**Keywords:** Regulatory landscape, Chromatin organization, Gene regulation, Topologically associating domains, TAD, Enhancer, *Hox*, CTCF, Limb development

## Abstract

**Background:**

The transcriptional activation of *HoxD* genes during mammalian limb development involves dynamic interactions with two topologically associating domains (TADs) flanking the *HoxD* cluster. In particular, the activation of the most posterior *HoxD* genes in developing digits is controlled by regulatory elements located in the centromeric TAD (C-DOM) through long-range contacts.

**Results:**

To assess the structure–function relationships underlying such interactions, we measured compaction levels and TAD discreteness using a combination of chromosome conformation capture (4C-seq) and DNA FISH. We assessed the robustness of the TAD architecture by using a series of genomic deletions and inversions that impact the integrity of this chromatin domain and that remodel long-range contacts. We report multi-partite associations between *HoxD* genes and up to three enhancers. We find that the loss of native chromatin topology leads to the remodeling of TAD structure following distinct parameters.

**Conclusions:**

Our results reveal that the recomposition of TAD architectures after large genomic re-arrangements is dependent on a boundary-selection mechanism in which CTCF mediates the gating of long-range contacts in combination with genomic distance and sequence specificity. Accordingly, the building of a recomposed TAD at this locus depends on distinct functional and constitutive parameters.

**Electronic supplementary material:**

The online version of this article (doi:10.1186/s13059-017-1278-z) contains supplementary material, which is available to authorized users.

## Background

Genes involved in key developmental processes are usually expressed in different tissues and at different times and hence they require particularly precise regulatory controls [1, 2]. To achieve this complexity in their transcription patterns, they often rely on the presence of multiple regulatory elements, including enhancer sequences (e.g., [3]). In addition, multiple enhancers can serve the same or a related specificity, either by acting as shadow enhancers to ensure robust transcription under adverse conditions [4–6], or by complementing one another in large regulatory landscapes to integrate and assemble various parts of one particular expression domain (e.g., [7]). In vertebrates, accumulating evidence suggests that most enhancers are located within such regulatory landscapes, often localized at some distance from the target gene(s).

The genome-wide application of chromosome conformation capture (e.g., [8]) has revealed the existence of topologically associating domains (TADs), an intermediate level of chromatin domains wherein enhancer–promoter interactions seem to be restricted and privileged [9–11]. TADs tend to be evolutionarily conserved [9, 12, 13] and are characterized by constitutive contacts and the involvement of chromatin architectural proteins. They can thus be observed in both transcriptionally active and inactive contexts. As a consequence, regulatory landscapes and their target gene(s) often overlap with TADs [14, 15]. However, the question as to whether TADs can restrict enhancer–promoter contacts or, conversely, whether enhancer–promoter contacts are instrumental in the building of a TAD remains to be clearly answered [2, 16].

Under physiological conditions, the interplay between multiple enhancers, constitutive contacts and both the nature and the extent of a particular TAD can be advantageously studied by using the *HoxD* gene cluster in embryo*. Hoxd* genes are transcribed in distinct combinations during embryonic development in a tissue- and time-specific manner, following their regulation by a series of cell-specific enhancers [7, 15, 17]. As in many other contexts, the identification of these regulatory sequences relied upon either particular histone modifications, chromatin accessibility, or the use of chromosome conformation capture (4C). A series of such long-range enhancers located in the centromeric TAD (hereafter referred to as C-DOM) are required during digit development to control the transcription of a set of target *Hoxd* genes, in particular *Hoxd13* [7, 18]. The targeted deletion of C-DOM almost entirely abrogated transcription in digits, whereas partial deletions gave intermediate outcomes, suggesting that these “regulatory islands” are all required to achieve the final and full transcription specificity [7]. However, the substitution of some regulatory islands by others through genomic rearrangements induced visible phenotypic consequences, indicating that these elements have specific features and cannot simply be inter-changed [19].

Here, we use this regulatory landscape to investigate whether various combinations of interactions between these enhancers and their target genes may occur in different cells (e.g., [20]). We also try to assess the importance of the genomic distance versus sequence specificity of these regulatory islands towards target genes by using a set of copy number variations (CNVs) including a series of nested deletions leading to important reorganizations of the C-DOM TAD. We report that the building of new TADs, after severe topological re-organization, depends on both the presence of characterized specific or constitutive interactions, including potential CTCF-driven contacts, as well as a relative distance effect, suggesting that intrinsic physical properties may also contribute to the shaping of these chromatin domains at this locus.

## Results

### The C-DOM TAD is a functional compartment for digit enhancer sequences

**Fig. 1:**
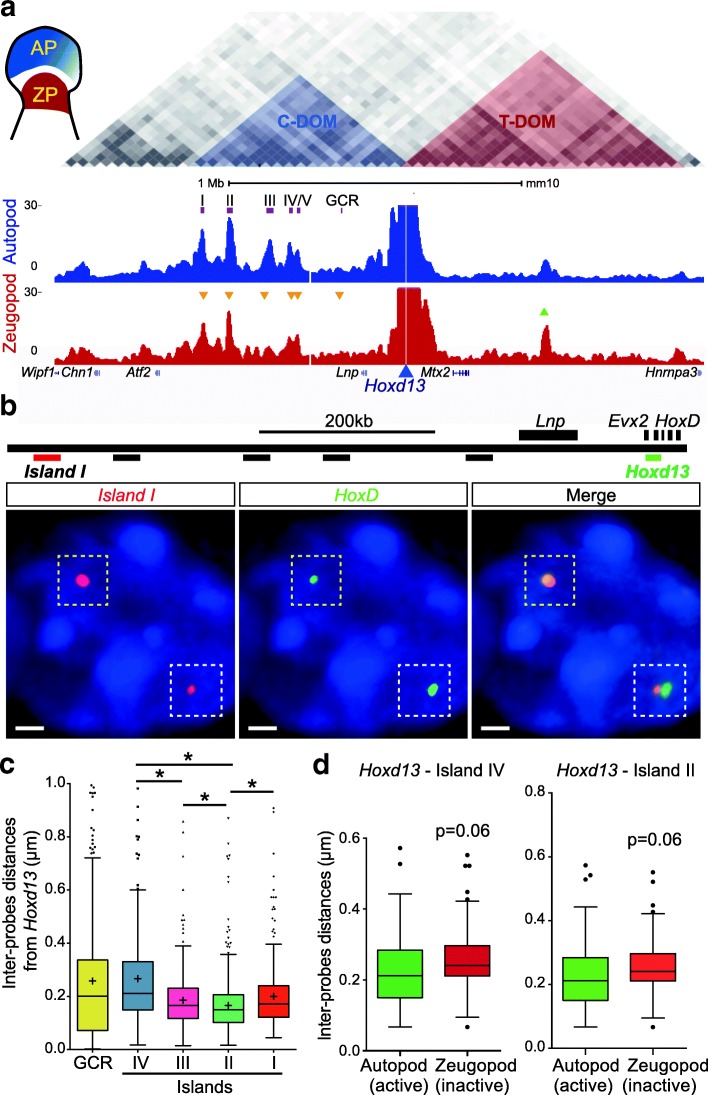
Interactions at the *HoxD* locus as seen by 3D DNA FISH and 4C-seq. **a** 4C interaction profiles (normalized signals) of *Hoxd13* in wild-type autopod (*AP*, *blue*) and zeugopod (*ZP*, *red*) cells, isolated from E12.5 embryonic mouse forelimb. The position of the TADs is highlighted on top (data from [9]). The triangles indicate statistically significant increases (*green*) or decreases (*orange*) in contacts on the main 4C peaks (*p* < 0.0001 using Wilcoxon rank test). **b** Positions of fosmid probes used for 3D DNA FISH. The images below show the FISH signals obtained for *Hoxd13* (*green*) and island I (*red*) as an example of both tightly associated signals (upper left allele, *yellow dashed square*) and separated signals (lower right allele, *white dashed square*). Scale bar: 1 μm. **c** Quantification of inter-probe distances between *Hoxd13* and the regulatory elements in autopods ranked *left* to *right*, from the closest to the furthest in terms of genomic distance. Kruskal–Wallis test was followed by Dunn’s multiple comparison tests: *p < 0.05. **d** 3D DNA FISH distances as measured in autopod (*Hoxd13* active, *green*) and zeugopod (*Hoxd13* inactive, *red*) cells from E12.5 mouse forelimbs. Both Tukey boxplot representations show shorter distances in those tissues where *Hoxd13* is active (Mann–Whitney test)

The C-DOM includes the core interactions between *Hoxd* genes and their digit enhancers, as defined by the interaction profile of *Hoxd13*, the main target of these enhancers within the gene cluster. To assess the dynamics of these interactions, we initially compared the contacts established by *Hoxd13* in both distal and proximal dissected limb bud cells. In distal autopod cells (presumptive digits), *Hoxd13* is transcribed robustly whereas in proximal zeugopod cells (presumptive arm), *Hoxd13* is inactive, thus allowing for a direct functional comparison as these distinct cellular domains share the same developmental origin (Fig. 1a). The examination of these 4C profiles (three different replicates) revealed the global map of *Hoxd13* contacts and allowed the identification of those interaction peaks that display the highest variation between cells where *Hoxd13* is active or inactive. In particular, contacts with islands I, II, III, and IV were increased in transcriptionally active distal cells. On the other hand, most contacts between *Hoxd13* and the telomeric TAD (T-DOM) appeared more robust in proximal cells, where the latter gene is transcriptionally inactive (Fig. 1a).

To evaluate whether such dynamic variations in interactions correlate with the position of the islands in the 3D space relative to *Hoxd13*, we performed 3D DNA FISH in distal and proximal cells (Fig. 1b) and found that, in these cells, most islands are located at a very short distance from *Hoxd13* (Fig. 1c). While such close associations were not unexpected due to the position of these islands within one TAD, we nonetheless noticed that the interaction peaks identified previously [7, 15] and in Fig. 1a as displaying a dynamic behavior were more closely associated with *Hoxd13* in distal limb bud cells where this latter gene is transcribed at high level. In particular, islands II and IV were significantly closer to *Hoxd13* in active cells when compared to inactive proximal cells (Fig. 1d).

The strongest associations revealed by 4C did not necessarily correspond to the closest genomic distances. Islands I and II, for instance, were often found within a 200-nm distance from *Hoxd13* whereas the GCR sequence, which is only weakly interacting by 4C (Fig. 1a) and island IV, was positioned further away (Fig. 1b, c). Other weak or non-interacting regions were observed at larger distances (Additional file 1: Figure S1). Such distance variations were significant (Mann–Whitney test) and well supported by the strong interaction peaks detected on islands I and II in distal cells (Fig. 1a; Additional file 1: Figure S2) [7, 15, 17, 21]. Likewise, the shortening in distance between islands I and II specifically observed in distal cells suggested that the regulatory landscape adopted globally more condensed configurations (Additional file 1: Figure S2), as shown for the entire C-DOM [22].

### Multipartite interactions

**Fig. 2:**
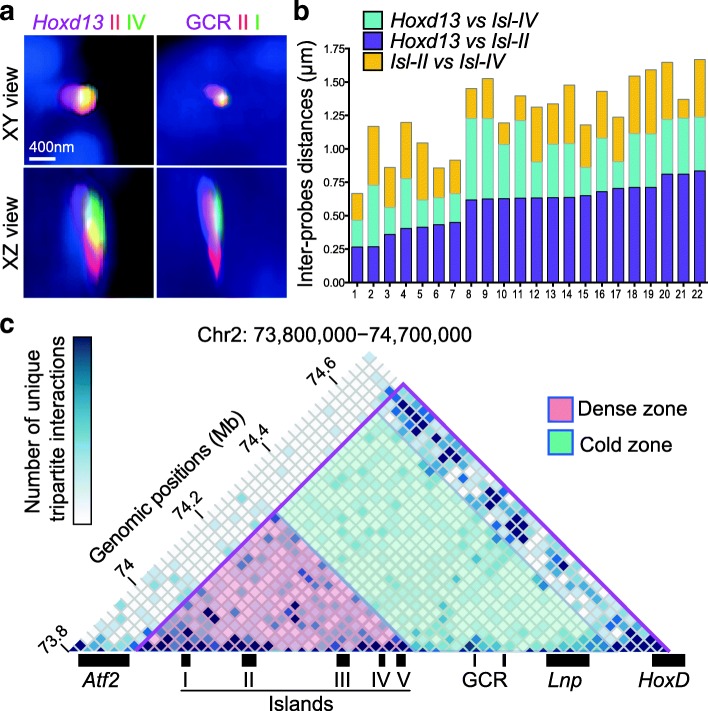
Tripartite interactions between *Hoxd13* and the regulatory islands. **a** Orthogonal projections from 3D DNA FISH (as in Fig. 1b) performed using either *Hoxd13* and two regulatory islands (*left*) or three islands (GCR, island II, and island I) in E12.5 forelimb autopod cells. Scale bar: 400 nm. **b** 3D DNA measurements showing variations in the distribution of physical distances between *Hoxd13* and island II (*purple*, *bottom*), *Hoxd13* and island IV (*cyan*, *middle*), or island II and island IV (*yellow*, *top*). **c** Heatmap of unique tripartite interactions in the centromeric TAD region generated from 4C-seq data using *Hoxd13* as a viewpoint (from *white*, corresponding to no interaction, to *dark blue*, indicating more than five unique interactions in the 20-kb bin). For each *Hoxd13*–X–Y triple interaction, the X and Y regions were assigned to a bin of 20 kb and reported into the matrix with bins of 20 kb. To avoid possible PCR artifacts, identical X–Y pairs were counted only once. Below are represented the positions of the genes and the digit enhancers. The *purple line* shows the limits of C-DOM as identified in [9]

The distribution of distances observed displayed a great heterogeneity from cell to cell. Furthermore, the extent of variation was slightly different for each regulatory island, suggesting that they may occasionally contact their target gene independently from one another. We tried to assess this issue by combining three probes in a DNA FISH experiment, specific for either three different islands or two islands as well as the *Hoxd13* target gene (Fig. 2a, b), and we observed several regulatory sequences juxtaposed in the same cell (Fig. 2a). These tripartite complexes, however, displayed heterogeneous configurations (Fig. 2b), despite the use of digit cells where the transcription of *Hoxd13* is supposed to be robust and homogenous [18]. Amongst the combinations scored, the occurrence of one island being located somehow at the interface between *Hoxd13* and another island was over-represented, suggesting that some of these genomic sequences may trigger the formation of larger regulatory structures.

Multiple and concomitant interactions can also be scored by using 4C-seq [23, 24] and we asked whether the complexes observed by DNA FISH could also be detected using 4C matrices (Fig. 2c). Interactions between two regulatory islands and *Hoxd13* were indeed detected and their frequencies were significantly higher in a more distant “hot zone” in the C-DOM TAD, where five regulatory islands are concentrated. In this region, 67% of 20-kb bins were detected in at least one tripartite interaction (Fig. 2c, red zone), whereas a cold zone was observed closer to the *Hoxd13* target, where only 39% of bins were involved in tripartite contacts (Fig. 2c, green zone). Altogether, these observations suggested that *Hoxd13* does not physically interact with all of its regulatory elements in every cell all the time. However, it can interact with multiple elements in the same cell at the same time.

### Regulatory versus genomic distances

**Fig. 3:**
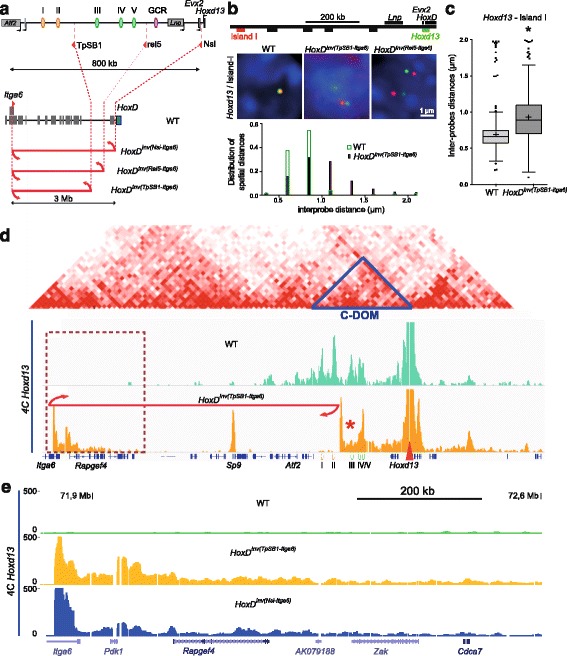
Impact of increasing distance on interactions with *Hoxd13*. **a** The large inversions displacing regulatory islands away from *Hoxd13*. **b** DNA FISH signals for *Hoxd13* (*green*) and island I (*red*) in distal limb bud autopod cells dissected from homozygous mutant embryos for either one of two large inversions. Scale: 1 μm. Below are shown the distribution of spatial distances in the wild-type (*WT*, *green*) and the inverted (*purple*) allele. **c**, Quantification of 3D distance showing the increase in distance observed in **b** (Mann–Whitney test, *p* < 0.001). **d** 4C-seq profile using *Hoxd13* as a viewpoint either in control (*green*) or the 2.4-Mb *HoxD* ^*Inv(TpSB1-Itga6)*^ inversion (*orange*). Above are aligned the heatmaps generated using data from [9] with C-DOM indicated by a *blue triangle*. The *Hoxd13* viewpoint is indicated with a *red arrowhead*. The breakpoints of the inversion are shown by the *red arrows*. The *red star* indicates the position of island III where contacts are lost in the inversion. **e** Close-up of region boxed in **d** where ectopic contacts are increased in both *HoxD* ^*Inv(TpSB1-Itga6)*^ and *HoxD* ^*Inv(Nsi-Itga6)*^ mutant samples

Altogether, these results indicated that some regulatory islands displayed higher interactions with the target gene than others, even though they are functional in the same cell type with the same specificity and irrespective of the genomic distance. Also, enhancers located within the same sub-domain tend to establish contacts with one another. To further investigate to what extent these two parameters influence the regulatory outcome, we assessed whether the relocation of some of these particular enhancer sequences outside C-DOM would modify contacts with the target gene. We used a large inversion where the two distal islands I and II were relocated 2.4 megabases (Mb) away (*HoxD* ^*(invTpSB1-Itga6*)^) [19] and analyzed the spatial distance between *Hoxd13* and island I by using 3D DNA FISH in autopod cells. We observed a clear loss of proximity as the distance between *Hoxd13* and island I was increased (Fig. 3b, c). This separation was similar to distances we had previously observed between other segments of DNA that do not form functional gene–enhancer contacts (Additional file 1: Figure S1) [25].

The 4C interaction profiles obtained in autopod cells using this large inversion showed a near complete absence of contact between either island I or island II with *Hoxd13*, confirming this increased distance (Fig. 3d) and suggesting that, to some extent, a minimal genomic distance may be required to allow and stabilize an interaction. However, increased contacts were observed in other instances over much larger distances—for instance, when using the 28-Mb *HOXD* ^*Inv(HoxDRVIII-Cd44)*^ inversion [26]. In this case indeed, a faint interacting region localized 19 Mb from the *HoxD* cluster (the *Alx4* gene promoter [25]) was re-positioned at a distance of 9.2 Mb from *HoxD*, leading to a visible decrease in distance by 3D DNA FISH (Additional file 1: Figure S3a, b) associated with a robust increase in the corresponding 4C signals (Additional file 1: Figure S3c).

In the case of the *HoxD* ^*inv(TpSB1-Itga6*)^ inverted allele, the loss of 4C contacts between island I and *Hoxd13* was compensated for by novel DNA contacts established between *Hoxd13* and the sequences relocated at the position of the displaced islands (Fig. 3d, e). These novel and ectopic contacts were discrete and of intensities comparable to those of known interactions normally occurring within this TAD. This result suggested that C-DOM had been re-organized into an interaction domain of a similar global size. This internal TAD re-organization, however, substantially impacted the contact dynamics of those islands that had not changed their genomic distances to the target gene, in particular islands III and IV, which displayed reduced peak sizes in the inverted allele (Fig. 3d, red star), whereas island V showed the opposite effect, giving rise to a global profile that resembled more that obtained with proximal, rather than distal, limb bud cells (Figs. 1a and 3d).

The extent of the contacts gained by *Hoxd13* with naive DNA sequences coming from the inversion nevertheless did not depend upon the genomic distance but instead involved some sequence specificity. Indeed, when we compared these additional contacts (Fig. 3) with those gained after using the *HoxD* ^*Inv(Nsi-Itga6*)^ allele, which inverted and thus re-localized the entire C-DOM [15] (Fig. 3e), we observed that in both cases the gained interactions span about 200 kb of the inverted DNA, with particularly strong peaks mapping on the promoter of *Rapgef4*, a gene that was brought to the vicinity of the *HoxD* locus by both inversions. Therefore, in both genomic rearrangements, this gene acted as a landmark in the building of new interaction domains, regardless of the overall sizes of these new domains.

### Impact of TAD rearrangement on transcription

**Fig. 4:**
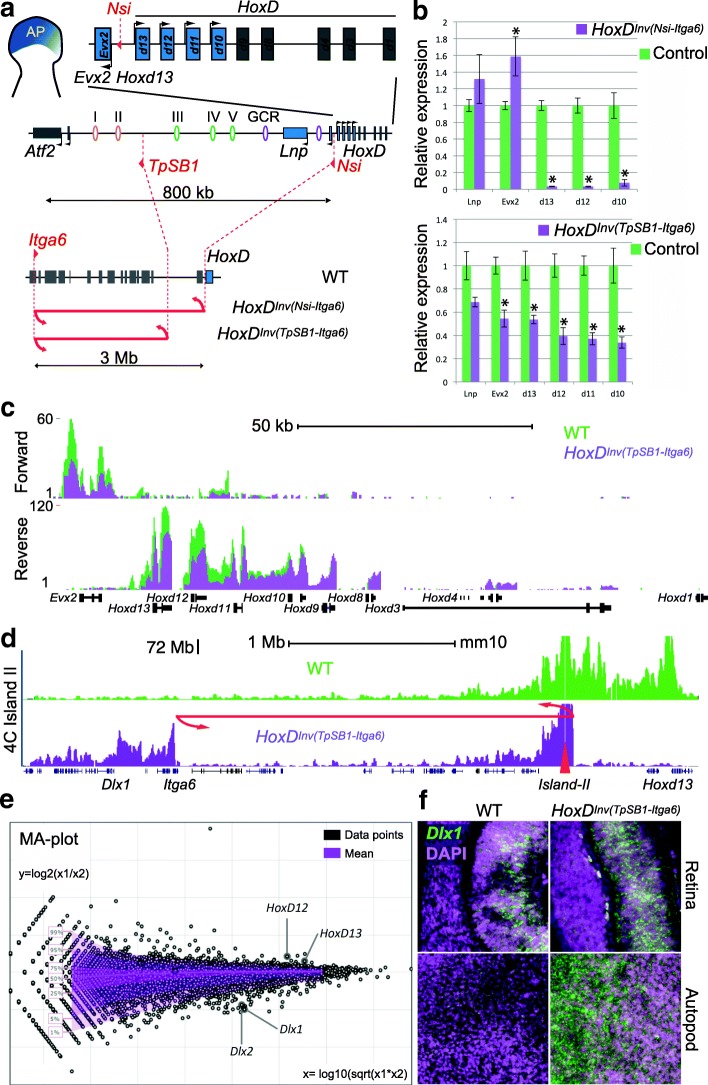
Impact of large inversions on transcription. **a** Two large inversions displacing regulatory islands away from *Hoxd13*. The *HoxD* cluster and the *Evx2* gene are shown on the *top* as well as C-DOM along with the position of the genes. The enhancers and the two breakpoints are depicted *below* in *red* (TpSB1, *left*; Nsi, *right*). At the bottom and using a different scale, the extent of the inversion is shown. **b** RT-qPCR quantification of target *Hoxd* gene mRNAs in presumptive digit cells either from the inversion mutant (*purple*) or from control (*green*) cells show a significant loss of *Hoxd10* to *Hoxd13* transcripts in both inversions. *Error bars* represent standard deviation (n = 3); *p < 0.05 (*t*-test). **c** Strand-specific RNA-seq profiles showing reduced transcription when the 2.4-Mb *HoxD* ^*Inv(TpSB1-Itga6)*^ inversion was used (*green*, wild type (*WT*); *purple*, inverted allele). **d** 4C-seq profiles using island II as a viewpoint (*red triangle*) in forelimb autopod cells from WT (*green*) and *HoxD* ^*Inv(TpSB1-Itga6)*^ inversion (*purple*) showing the gain of contacts with the *Dlx1* locus in the inversion allele. **e** Changes in expression as measured by RNA-seq are represented as a MA plot, with *Hoxd12* and *Hoxd13* down-regulated in the inversion (as in **b**) whereas *Dlx1* and *Dlx2* were up-regulated. *Dlx1* up-regulation is controlled by RNA FISH in **f**, with the upper panels showing the retina and the lower panel distal limb autopod cells (both in E12.5 embryos)

To assess whether these recomposed TADs were still able to properly regulate their *Hoxd* target genes, we measured *Hoxd* genes expression using RT-qPCR in autopod cells (Fig. 4a-b). All *Hoxd* genes previously shown to interact with C-DOM [7] displayed somewhat reduced steady-state levels of mRNAs (Fig. 4b). We used RNA-seq to evaluate the transcription of genes located in *cis* of the *HoxD* cluster and could confirm the decreased transcription of posterior *Hoxd* genes in these re-organized genomic topologies (Fig. 4c). In addition, we detected a set of up-regulated genes (Additional file 1: Table S1), including *Dlx1* and *Dlx2*, two genes located a few megabases from the *HoxD* cluster and neighboring islands I and II following the inversion (Fig. 4d, e). We confirmed the apparent transcriptional up-regulation of *Dlx1* by RNA FISH and observed a clear increase in signal intensity specifically in distal forelimb cells. In cells from the retina, a tissue where *Dlx1* is already expressed [27] but where the regulatory islands I and II are normally not functional, this transcriptional boost was not detected (Fig. 4f).

### Pre-determined reallocation of contacts

**Fig. 5:**
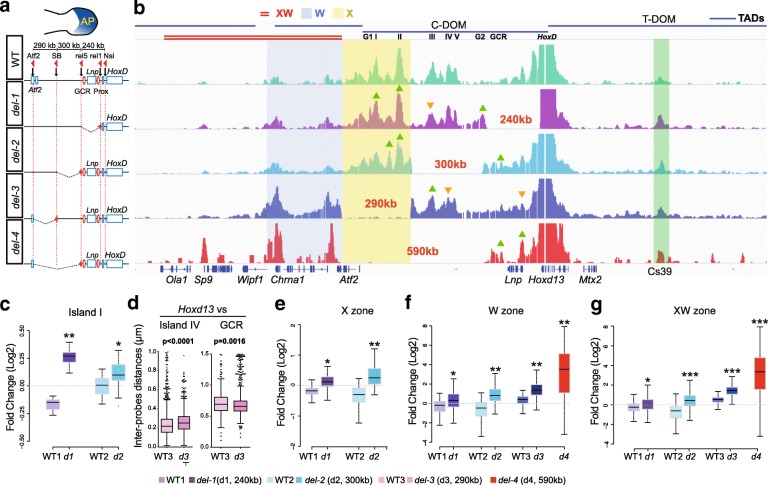
Reallocation of long-range contacts in reorganized TADs. **a** Forelimb bud with the autopod in *blue* is shown *above* the deletion series with the various positions of loxP site used to engineer the deletions (*red arrowheads*). **b** 4C interaction profiles from autopod cells with *Hoxd13* as a bait in control (wild type (*WT*), *green*), *del-1* (*purple*), *del-2* (*light blue*), *del-3* (*dark blue*), and *del-4* (*red*). The four *horizontal bars* on *top* represent the TADs as taken from [9]. The position of the islands is indicated below the bar of C-DOM. The X zone is highlighted in *yellow*, the W zone is highlighted in *blue*, and the *double red line* on *top* indicates the XW zone. The *triangles* indicate statistically significant increases (*green*) or decreases (*orange*) in contacts (*p* < 0.05 using Dunn test using *p* value adjustment with the Benjamini–Hochberg procedure). **c**, **e**–**g**, The Y-axis represents a log2 scale. **c** Fold change on specific contacts as observed in **b** for island I. **d** Distance measurements from 3D DNA FISH between *Hoxd13* and island IV (*left*) and GCR (*right*) in autopod cells from WT and *del-3* specimens. **e** Fold change in “region X” (highlighted in *yellow* in panel **b**). **f** Fold change in the “W zone”, highlighted in *blue* and centromeric to all deletions. **g** Fold change in the “XW zone”, a 590-kb region (highlighted in *red*) containing the W zone and extending further centromeric to all four deletions. *p < 0.0001; **p < 10^-30^; ***p < 10^-80^ (compared with a Wilcoxon rank sum test)

The interactions observed between *Hoxd13* and the DNA relocated after the large inversion suggested that genomic distance represents a physical constraint in the building of the C-DOM TAD structure. Alternatively, this inversion may have led to the redistribution of boundary elements, leading to a novel TAD. To address this issue, we used a series of deletions internal to C-DOM in order to modify internal genomic distances (Fig. 5, *del-1* to *del-4*). We compared the 4C profiles of the various deleted configurations in autopod cells (Fig. 5; Additional file 1: Figure S4) and noted a high conservation of sequence-specific interactions, regardless of which internal segment of the TAD had been removed (Fig. 5b). Contacts persisted on the remaining islands independent of the change in genomic distances, indicating that the global TAD structure in itself does not seem to be required for all internal contacts to be optimally established.

In the *del-1* allele, shortening the distance between the islands and their target genes by 240 kb had little effect on their interaction profiles (Fig. 5b). Statistical analyses, however, revealed that when islands I and II were positioned at the places of islands III and IV through the *del-1* deletion, their interaction peaks increased in intensity (Fig. 5c; Additional file 1: Figure S4c). Instead, the island III peak, which was shown to be specific to digit cells [15], was reduced even though located closer to its interacting genes in terms of genomic distance (Additional file 1: Figure S4d). Such slight modifications in peak intensities were also observed in the *del-2* and *del-3* deletions (Fig. 5b, green and orange arrowheads, and Fig. 5c; Additional file 1: Figure S4c, d; log2 fold change compared to their wild-type littermates).

Of note, whenever a particular deletion induced the strong re-enforcement or even the appearance of an interaction peak not detected in control cells, this sequence usually corresponded to a region of specific interaction in another cellular context or, alternatively, in another TAD. The first case was best illustrated by the *del-1* allele, where interactions involving *Hoxd13* were increased with the GT1 and GT2 sequences (Additional file 1: Figure S4c, d). These two enhancers normally interact with this gene but only in the developing genitals and not in developing digit cells [17]. They were, however, recruited in the novel regulatory topology induced by this particular deletion.

The second case was observed with the *del-3* and *del-4* mutant alleles. The *del-3* deletion triggered *Hoxd13* to establish two major contacts upstream of its usual proximal TAD boundary, within what is the adjacent TAD in the wild-type situation (Fig. 5b, region W). These two interactions peaks, however, could already be observed in the control and *del-1* and *del-2* profiles, where the TAD boundary is not deleted, though with a much lower intensity. Therefore, deletion of the TAD border merely reinforced the contacts, which were already established yet at a much lower frequency (Fig. 5f). This was supported by the largest internal *del-4* deletion, which removed regulatory islands I to V. In this case, the severely decreased expression of *Hoxd13* reported for this configuration [7] correlated well with the robust interaction with the *Sp9* gene. Indeed, these loci were shown to interact with one another when transcriptionally inactive [25]. However, weak interactions with this gene were already detected in control cells, suggesting that these interactions were not fully de novo induced by either reducing the distance or deleting a TAD boundary. However, the contacts extended further in the surrounding region XW until the gene *Ola1* (Fig. 5b).

In the *del-3* allele, we noted that the strong increase in contacts in region W occur even though the genomic distance is the same as in the *del-2* allele, where such gains of contacts were not observed. This was likely due to the removal of the proximal TAD boundary in the *del-3* allele, whereas this boundary is still present in the *del-2* chromosome. In this view, the TAD boundary seems to be important to properly assign interaction strengths to contacts that normally occur, sometimes at very low frequencies. The strong distal interactions in *del3*, leading to what appears to be a fused TAD including C-DOM and region W (Fig. 5b), also slightly re-organized contacts occurring in the proximal part of the TAD, such as an increase in contacts with island III and a decrease with island IV. However, this re-organization involved sequences already interacting, rather than de novo contacts (Fig. 5b).

Finally, any genomic modification of the C-DOM landscape led to an increase of contacts between *Hoxd13* and T-DOM, the TAD located at the opposite side of the gene cluster. This was particularly visible with the Cs39 region, an enhancer active in proximal cells during the development of the zeugopods [15, 28] (Fig. 5b, green bar). However, this region constitutively contacts *Hoxd13* at low levels (Fig. 1a; Fig. 5b, upper 4C profile). This observation suggests that the optimal topological configuration to transcribe *Hoxd13* in digit cells involves the presence of a native C-DOM, as all deletions tested lead to decreases in mRNA levels. In this optimal situation, *Hoxd13* is fully engaged in interacting with the centromeric islands. Whenever a genomic perturbation is induced, *Hoxd13* loses some interactions with C-DOM and thus can be re-directed towards the major T-DOM interactions, concomitant with a change in transcriptional outcome.

### Boundary effects

In the *del-4* allele, where all five islands were deleted, not only did the contacts extend into the neighboring TAD (Fig. 5b, f, zone W) but they were also detected up to a distance similar to the genomic segment that had been deleted (Fig. 5b, g, zone XW), thereby reaching the *Sp9* gene and behind. In the *del-1* and *del-2* alleles, however, the extension of the interactions towards the centromeric side importantly decreased at the *Atf2* gene. In the *del-3* allele, on the other hand, contacts increased up to the *Chrna1* gene yet not really beyond (Fig. 5b). These observations may reflect the presence of a potential TAD boundary as previously mapped by Hi-C [9]. Indeed, the *del-1* and *del-2* alleles did not physically affect such a boundary, whereas the *del-3* allele removed the TAD boundary between C-DOM and the TAD included in region W, allowing for *Hoxd13* contacts to extend into this latter TAD.

However, the results obtained with the *del-4* allele could not be interpreted similarly, for no additional boundary was deleted when compared to *del-3*, yet the interaction profile largely extended into the centromeric region, reaching the *Sp9* and *Ola1* gene bodies (Fig. 5b). Also, in the two large inversions discussed above (Figs. 3e and 4a) where the C-DOM TAD boundary was removed in both cases, the contacts extended up to the same gene, *Rapgef4*. Because *Hoxd13* is located close to a TAD boundary and is surrounded by CTCF sites [29], which are closely associated with TAD boundaries [9, 30–32], we looked at the presence of bound CTCF in these various regions to address these differences.

**Fig. 6:**
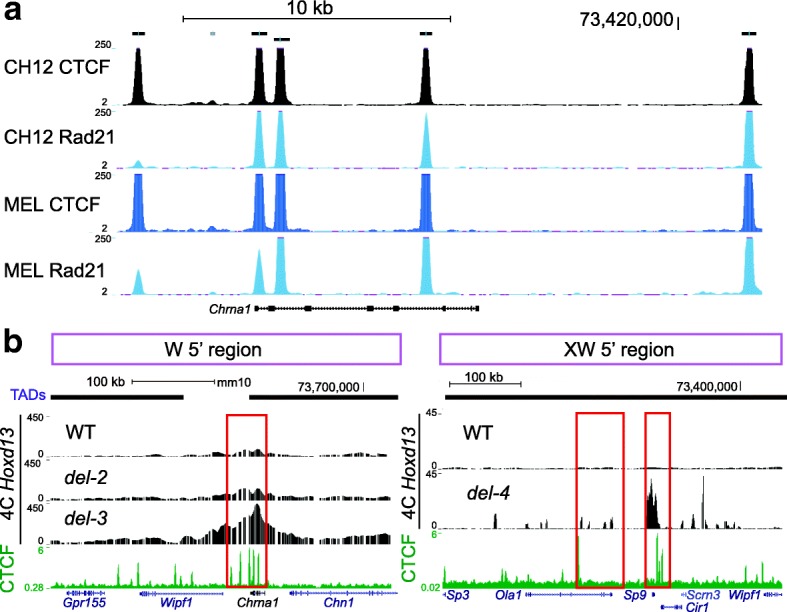
De novo TAD boundaries are CTCF-rich regions. The tracks corresponding to the CTCF and cohesion Chip-seq data were extracted either from ENCODE (**a**) or from our experiments using distal limb bud cells at E12.5 (**b**). They are displayed in the UCSC Genome Browser (https://genome.ucsc.edu/cgi-bin/hgTracks?db=mm9&lastVirtModeType=default&lastVirtModeExtraState=&virtModeType=default&virtMode=0&nonVirtPosition=&position=chr2%3A73563281-73580338&hgsid=601487871_7lJaNpYWWLpaaX1lmfxZSqC4FLKC). The regions emphasized are the DNA segments where contacts drastically decrease, as observed in Fig. 5b. *WT* wild type

We performed ChIP-seq for CTCF using E12.5 autopod cells and confirmed the binding pattern previously reported by a ChIP-on-Chip approach using the same embryonic tissue [29]. We also compiled the ENCODE data for CTCF and the cohesin subunit RAD21 and all datasets revealed five CTCF peaks surrounding the *Chrna1* locus, with four being also bound by cohesin (Fig. 6a, b). This further qualifies this region as a TAD boundary and thus explains the re-organized interaction profiles observed with the *del-3* alleles as well as the weak contacts already detected between *Hoxd13* and this region in the *del-1* and *del-2* alleles.

Multiple sites bound by CTCF were also scored at the *Sp9* locus (three peaks) and near *Ola1* (one peak), despite the fact that this zone was never reported to be a TAD boundary (Fig. 6b). Finally, we observed a comparable enrichment of CTCF peaks (seven or more) in both the promoter and the *Rapgef4* gene body (not shown). Therefore, while it seems that, under our physiological conditions, the reorganization of topological domains after targeted copy number variation will generally involve the use of bound CTCF as novel landmarks to exert a boundary effect, exceptions exist, such as the *del-4* allele, where the *Chrna1* locus, a strong boundary element, is ignored and interactions established between *Hoxd13* and *Sp9*, which itself lies in the middle of another TAD.

## Discussion

### Multiple interactions

Our 4C-seq experiments identified multiple contacts between more than two sequences at a time, suggesting that the overall interaction profile observed by using millions of cells may represent, at least in part, what is happening within single cells. It nevertheless does not allow us to propose any conclusion regarding the overall dynamics of contacts within this particular C-DOM, i.e., whether the increased occurrence of particular multiple contacts is due to a longer interaction time or, alternatively, to a higher frequency of contacts between the sequences involved. Therefore, while these results suggest that some contacts may be cooperative or perhaps requested to properly bring an enhancer in the vicinity of its target gene, the number of potential TAD configurations and their respective frequencies [20] cannot be predicted. Tripartite interactions at this locus were also studied recently in embryonic stem cells where transcription is not observed and may thus reflect a purely constitutive state [33]. In that study, however, the tripartite interactions were more homogenous and the cold zone was less defined than in digit cells.

This cold zone was identified in our tripartite 4C matrices and corresponds to a region of low contacts when using the *Hoxd13* locus as a viewpoint. This was confirmed by our FISH data, where a sequence falling within this space, the GCR, showed the highest mean distance to *Hoxd13* (Fig. 1c). Also, a previous serial deletion analysis in vivo had suggested that each part of this TAD was functionally rather independent from the others [7]. Altogether, by using several parameters, the TAD sub-region covering islands I to V appeared more dense and structured than the region immediately flanking the *HoxD* cluster in transcriptionally active distal limb cells.

### Contact re-allocations

Of note, out of the numerous analyzed mutant alleles that re-compose the genomic topologies at this locus, none of them produced a strong interaction that had not been observed previously either in a control or in another mutant, nor did they radically suppress any existing interaction. For example, when large inversions were used, none of the contacts left between *Hoxd13* and the remaining parts of the TAD disappeared, despite the re-composition of the structure due to the addition of foreign DNA (Fig. 3). Likewise, when various deletions including parts of the TAD were analyzed, none of the remaining interactions were seriously affected, even when large deletions were used. The *del-2* allele, for instance, removed three strong points of contacts with *Hoxd13* (islands III, IV, and V), yet the interaction profile of *Hoxd13* with the remaining islands I and II was almost as in the control limbs. We take this as a strong indication that individual contacts within a TAD have only a moderate impact upon the global architecture of the TAD.

An exception to this was the behavior of island III, a *Hoxd13*-interacting sequence exclusively detected in developing digits (e.g., [28]). In the *del-1* allele where island III became closer to the cluster as a result of the deletion, its interaction with *Hoxd13* was importantly diminished (Fig. 5b; Additional file 1: Figure S4d). This effect was also observed in the large inversion where both islands I and II had been displaced (Fig. 3d). In these genomic contexts, the loss of island III contacts paralleled the loss of activity of *Hoxd13*, suggesting that part of the functional impact of these re-organizations was due to *in cis* effects on non-deleted sequences, rather than solely to the deleted sequences themselves.

We also noted the appearance, in the *del-1* context, of the two GT1 and GT2 interaction peaks, which are not observed in control digit cells but are present in the developing genitals, where *Hoxd13* is expressed equally strongly (Fig. 5b; Additional file 1: Figure S4) [17, 34]. Therefore, the re-organization of the topological domain resulted in the recruitment of sequences that are used in a different context to control the same target gene. These results suggest that potential sites of interactions are fixed, but that tissue-specific TAD architectures can select a defined subset thereof in a given regulatory context. Unfortunately, we could not directly assess whether these two sequences actively participated in the transcriptional outcome of the domain or, alternatively, whether these contacts were purely induced by the new topology adopted after the *del-1* deletion without any particular effect.

### TAD modularity

The various regulatory islands do not seem to require a domain structure to exert their functional potential. They need not be embedded into a TAD to properly work, as shown by both deletion and transgenic analyses [7, 17], suggesting that TADs at *Hox* loci have a modular structure. This was confirmed by functional analyses of some of the mutant configurations used in this work. For example, the transposition of islands I and II close to the *Dlx1/Dlx2* locus outside of their “native” TAD lead to the up-regulation of these two genes in a domain where they are normally not transcribed. Such regulatory side effects [35] can often be observed after large *in cis* [36, 37] or *in trans* [38] genomic re-arrangements. As the *Dlx1*-*Dlx2* locus is normally covered by polycomb complexes in this cell type [28], the contacts with enhancer sequences may help to evict these repressive complexes, as described in the globin system [39]. Aberrant transcriptional outcomes deriving from such CNV-dependent enhancer–promoter re-allocations are likely to cause genetic diseases in a variety of instances [37, 40, 41].

### TAD boundaries, CTCF, and pre-set interactions

The analyses of our mutant genomes where the centromeric *HoxD* TAD was re-configured in different ways revealed two trends, which may reflect more general properties of chromatin folding. The first is that whenever strong interactions were gained between *Hoxd13* and sequences located outside the C-DOM as a result of a deletion, these sequences already displayed some (very) weak interactions in the control situation. Therefore, ectopic contacts were not de novo interactions but rather the re-enforcement of pre-existing weak interactions established despite the presence of the original TAD boundary. The deletion of such a TAD boundary (for example, in the *del-3* allele) induced a strong leakage of *Hoxd13* interactions but specifically towards sequences already weakly contacted in control cells. We take this as an indication that the contact map of a particular gene is likely independent of its TAD environment. In this view, TADs would impose a bias on high-affinity contact distribution by favoring local interactions within a spatial framework over outside contacts.

The re-organization of TADs often involves the presence of architectural proteins such as CTCF and cohesin [37, 42, 43]. Here again, either in the *del3* allele or in our large inversions, the ectopic contacts were gained up to a region rich in such proteins, as in the case of *del-3* where *Hoxd13* interactions could extend up to the *Chrna1* locus, which acted as a new TAD boundary. However, in the *del-4* configuration (a deletion larger than *del-3* but containing the same boundary elements), contacts extended beyond the *Chrna1* locus even though it was bound by CTCF at five positions, four of which also contained cohesin, to reach *Sp9*, another locus with bound CTCF. In this context, increased contacts with Sp9 may also be facilitated by the H3K27 trimethylation of the inactive *Hoxd13* and thus likely reflects polycomb-associated contacts [25]. We consider this an indication that boundary elements are not sufficient to impose a TAD structure and that other parameters may be equally important in shaping chromatin at this structural level. This latter result, along with our observations on the two large inversions, suggests that the physical distance may be important, as TAD re-organization at least at this locus tends to generate interaction profiles of rather comparable sizes, perhaps reflecting intrinsic forces or constraints at work at this scale of the chromatin fiber. This may support the idea, based on FISH data, that the chromatin fiber has a random-walk configuration but confined within a defined volume in the range of megabases [44–46].

## Methods

### Animals

Mice were raised and sacrificed according to good laboratory practice standards. Tissues were isolated from E12.5 embryos, either wild type or mutant for four different deletions as well as two large inversions. The deletions were *HoxD* ^*Del(rel1-rel5)*^ (referred to as *del-1* throughout the paper), *HoxD* ^*Del(rel5-TpSB1)*^ or *del-2*, *HoxD* ^*Del(TpSB1-Atf2)*^ or *del-3*, and *HoxD* ^*Del(rel5-Atf2)*^ or *del-4* [7]. The inversions were *HoxD* ^*Inv(TpSB1-Itga6)*^ and *HoxD* ^*Inv(Nsi1-Itga6)*^ [19]. All deletions and inversions were analyzed by using tissues dissected from homozygous embryos.

### Statistical analysis

For DNA FISH analyses, the differences between samples were evaluated with the Kruskall–Wallis test, followed by Dunn’s multiple comparison post test. For the 4C-seq box plots (Figs. 1 and 5; Additional file 1: Figure S4), the statistical differences between islands and regions of interest (islands, GCR, *Chrna1*, and the three zones described) were assessed by using pairwise Wilcoxon rank sum tests, followed by Benjamini–Hochberg corrections for multiple testing (false discovery rate) [47].

### 3D DNA FISH

3D DNA fluorescent in situ hybridization was as previously described [25, 48]. E12.5 mouse embryos were fixed in 4% paraformaldehyde, embedded in paraffin blocks, and cut at 6 μm. Sections were oriented such that cells belonging to either the distal (autopod) or proximal (zeugopod) parts of the growing limb bud could be unambiguously identified. Probes were prepared by nick-translation with directly labeled nucleotides (Ulysis alexa 647, Life Technologies) biotin- or digoxigenin-UTP using fosmid clones obtained from the BACPAC Resources Center (https://bacpacresources.org) and listed in Additional file 1: Table S2. DNA (100 ng) was used with 7 μg of Cot1-DNA and 10 μg of sonicated salmon sperm DNA. They were labeled using either digoxigenin- or biotin-dUTP by nick translation with fluorescent revelations as described in [48], using either Alexa 647, Alexa 568, or Alexa 488 as fluorophores. Slides were stained with DAPI and mounted in ProLong Gold (Life Technologies). Images were acquired using a B/W CCD ORCA ER B7W Hamamatsu camera associated with an inverted Olympus IX81 microscope. The image stacks with a 200-nm step were saved as TIFF stacks. Image reconstruction and deconvolution were performed using FIJI (NIH, ImageJ v1.47q) and Huygens Remote Manager (Scientific Volume Imaging, version 3.0.3). Distance measurements between probe signals were determined using an automated spot/surface detection algorithm followed by visual verification and manual correction using IMARIS version 6.5, Bitplane AG, and Matlab 7.5, MathWorks SA. Data from Fig. 1 were evaluated using only manual measurements. Statistical significance analyses of distances were performed using Mann–Whitney test (Figs. 1d, 3c, 5d; Additional file 1: Figures S2b and S3b), or using the Kruskal–Wallis test followed by Dunn’s post test (Fig. 1c). The images can be found on Figshare (Fig. 1b, doi:10.6084/m9.figshare.5198161; Fig. 2a, doi:10.6084/m9.figshare.5198242; Fig. 3b, doi:10.6084/m9.figshare.5198326; Additional file 1: Figure S3a, doi:10.6084/m9.figshare.5198386).

### RNA FISH

E12.5 distal limbs were fixed in 4% paraformaldehyde, 15% sucrose and then frozen in OCT. Cryostat sections (25 μm) were dried for 30 minutes, post-fixed in 4% paraformaldehyde for 10 minutes, and quenched with 0.6% H_2_O_2_ in methanol for 20 minutes. Slides were then processed using the Ventana Discovery xT with the RiboMap kit. The pretreatment was performed with mild heating in CC2 for 12 minutes, followed by protease3 (Ventana, Roche) for 20 minutes at room temperature. Finally, the sections were hybridized using an automated system (Ventana) with a *Dlx1* probe diluted 1:1000 in ribohyde at 64 °C for 6 h. Three washes of 8 minutes in 2× SSC followed at a hybridization temperature of 64 °C. Slides were incubated with anti-DIG POD (Roche Diagnostics) for 1 h at 37 °C in BSA 1% followed by a 10-minute revelation with TSA substrate (Perkin Elmer) and 10 minutes in DAPI. Slides were mounted in ProLong fluorogold. Images were acquired using the same procedure as for DNA FISH. The images can be obtained on Figshare (doi:10.6084/m9.figshare.5198359).

### RNA-seq

E12.5 distal forelimbs were dissected and isolated using Trizol LS reagent (Life Technologies) to generate total RNA tissue samples. RNA-Seq was performed according to the TruSeq Stranded Illumina protocol, with poly(A) selection. The strand-specific total RNA-seq libraries were constructed according to the manufacturer’s instructions (Illumina). Sequencing was done using 100-bp single-end reads on the Illumina HiSeq system according to the manufacturer’s specifications. RNA-seq reads were mapped to ENSEMBL Mouse assembly NCBIM37 and translated into reads per gene (RPKM) using the RNA-Seq pipeline of the Bioinformatics and Biostatistics Core Facility (BBCF) HTS station (http://htsstation.epfl.ch). RNA-seq data can be found in the Gene Expression Omnibus (GEO) repository under accession number GSE98232.

### 4C-seq

Micro-dissected E12.5 proximal or distal limb bud tissues were dissociated, fixed with 2% formaldehyde, lysed, and stored at −80 °C. The nuclei from ten pairs of distal or proximal forelimbs were then digested with a sequence of NlaIII and DpnII, followed by amplification according to [49]. The ligation steps were performed using high concentrated T4 DNA ligase (M1794, Promega) and the inverse PCRs for amplification were carried out using primers specific for the various viewpoints [50]. *Hoxd13* amplification primers were previously described [50]. PCR products were multiplexed and sequenced with a 100-bp single-end Illumina HiSeq flow cell. Demultiplexing, mapping to the mouse assembly GRCm38 (mm10), and 4C-Seq analysis were performed using the BBCF HTSstation (http://htsstation.epfl.ch and [51]), according to [50]. Briefly, the 4C-Seq fragments directly surrounding the viewpoints (2 kb) were excluded for the rest of the analysis. Fragment scores were normalized to the total number of reads mapped and smoothed (running mean with a window size of 11 fragments). For comparison purposes, the 4C-seq profiles were normalized to the mean score of fragments falling into a region defined as the bait coordinates plus or minus 1 Mb. In Fig. 1, a profile correction method similar to that described in [52] was applied to emphasize the relevance of long-range interactions with the islands. This was done using a fit with a slope −1 in a log-log scale [53]. For quantification of 4C-seq profiles in specific islands or regions of interest (box plots in Additional file 1: Figure S2 and Fig. 5), the smoothed data, with or without profile correction, were used. When appropriate (e.g., signals in Fig. 1b), replicates were combined by averaging the resulting signal densities. In Fig 5 and Additional file 1: Figure S4, quantitative log2 ratios were calculated by dividing the fragment scores with the means in WT1, WT2, and WT3. The number of mapped reads for each sample were as follows: in Fig. 1 (wild type only), 19,160,126 for autopods and 13,257,140 for zeugopods; in Figs. 3 and 4 (inversions) 9,050,395 for *Hoxd13* and 4,134,080 for island II; in Fig. 5 (deletion series), 3,722,913 for WT1, 3,812,342 for del1, 8,943,042 for WT2, 6,112,348 for del2, 6,494,171 for WT3, and 6,702,116 for del3. 4C-Seq data are available from the GEO repository under accession number GSE98861. In order to detect tripartite interactions, one of the 4C libraries was re-sequenced as 250-bp single-end. The reads were de-multiplexed using fastx_barcode_splitter (http://hannonlab.cshl.edu/fastx_toolkit/) and the viewpoint sequence was removed except for CATG (first cutter sequence) with seqtk (https://github.com/lh3/seqtk). Then they were trimmed for low quality and the presence of GATC (second cutter sequence) with cutadapt (cutadapt -q 10 -a GATC) [54]. Next, they were split if a CATG was present. The 5′ part of the split reads (hereafter referred to as “mid”) and the 3′ part of the split reads (hereafter referred to as “third”) were mapped independently with bowtie [55] (version 0.12.9) on mm10 (bowtie -p 5 -S -k 1 -m 1 -I 0 --best –strata). If the third read did not map, they were split again for CATG and only the first part was now considered as third and mapped. Reads for which the mapping of mid and third were consecutive (undigested situation) were not considered in the analysis. The reads were then pooled according to the mapping position and strand of the mid and the mapping position and strand of the third to remove potential PCR duplicates, resulting in a list of unique tripartite interactions. Each tripartite interaction in the 73.8–74.7 Mb region of chromosome 2 was assigned to a 20-kb bin and the matrix showing the number of different tripartite interactions was plotted.

### CTCF ChIP-seq

A total of 100 mg of distal limbs were dissected from wild-type CD1 embryos at E12.5 and fixed for 10 minutes in 1% paraformaldehyde solution. Fixation was stopped with glycine (0.1 M final concentration) and the pellet was washed three times with PBS and stored at −80 °C. Chromatin extraction and immunoprecipitation were performed using the ChIP-IT High sensitivity kit (Active motif) according to the manufacturer’s specification with slight modifications. Nuclei were extracted and sonicated in 600 μl of sonication buffer (50 mM Tris pH = 8.0, 1% SDS, 10 mM EDTA) using a Vibra Cell tip sonicator to obtain fragments with an average size of 200–300 bp. Subsequently, 25 μg of sonicated chromatin were diluted ten times in ChIP dilution buffer (20 mM HEPES, 150 mM NaCl, 0.1% NP40) and incubated overnight at 4 °C with 4 μg of anti-CTCF antibody (active motif) on a rotating platform. The next day chromatin–antibody complexes were incubated with protein A/G agarose beads for 3 h at 4 °C and successively washed following the manufacturer’s instructions. Finally, they were eluted and purified by phenol:chlorophorm extraction and precipitation. A total of 20 ng of immunoprecipitated DNA was sequenced with a 50-bp single-end Illumina HiSeq flow cell. Sequenced reads were mapped against the mouse GRC38/mm10 genome assembly using the BBCF HTSstation (http://htsstation.epfl.ch) [51] platform. The dataset were deposited in GEO (accession number GSE96558).

### RT-qPCR

RNA was extracted from pools of micro-dissected limbs or parts thereof with the Qiagen Tissue Lyser and Qiagen RNeasy Plus kit. RNA (500 ng) was reverse transcribed using random hexamers and Superscript III (Invitrogen). Relative and absolute qPCR were performed with 1 ng of template in technical triplicate. Primers and protocols were described in [18].

## Additional file

Additional file 1: Figures S1–S4 and **Tables S1** and **S2**. (PDF 1589 kb)
