# Supplementary material for: Large scale genomic reorganization of topological domains at the HoxD locus
Source: Genome Biol. 2017 Aug 7;18:149. doi: 10.1186/s13059-017-1278-z (PMC5547506; doi:10.1186/s13059-017-1278-z)
Supplement: Additional file 1: Figures S1–S4 — and Tables S1 and S2. (PDF 1589 kb) [file 13059_2017_1278_MOESM1_ESM.pdf]

Title:

LARGE SCALE GENOMIC REORGANIZATION OF TOPOLOGICAL DOMAINS AT THE *HoxD* LOCUS

Running Title: TAD formation at the *HoxD* locus

Authors:

Pierre J. Fabre[1], Marion Leleu[1], Benjamin H. Mormann[1], Lucille Lopez-Delisle[1], Daan Noordermeer[1,3] Leonardo Beccari[2] and Denis Duboule[1,2,*]

ADDITIONAL FILE 1

Supplementary Figures 1 to 4 with legends

Supplementary tables S1 and S2

Supplementary Figure S1

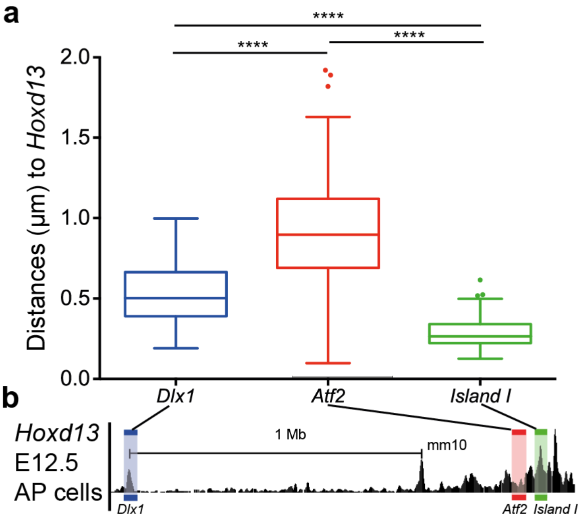

Figure S1. FISH distances are inversely correlated to the peak intensity as observed in 4C.
a. Quantitation of DNA-FISH signals showing inter-probe distances between *Hoxd13* and either a strong interacting region (island I), a weak interacting region (*Dlx1*) or a non-interacting region (*Atf2*). Kruskal-Wallis test was followed by Dunn's multiple comparison test: **** $p<0.0001$. Numbers of pairs analyzed are: 197 (*Dlx1*), 159 (*Atf2*) and 139 (island I). **b**. 4C interaction profiles (normalized signals) of *Hoxd13* in wild-type autopod cells, isolated from E12.5 embryonic mouse forelimb. The position of the three FISH probes used in **a** for *Dlx1* (blue), *Atf2* (red) and island I (green) are shown above the 4C profile.

Supplementary Figure S2

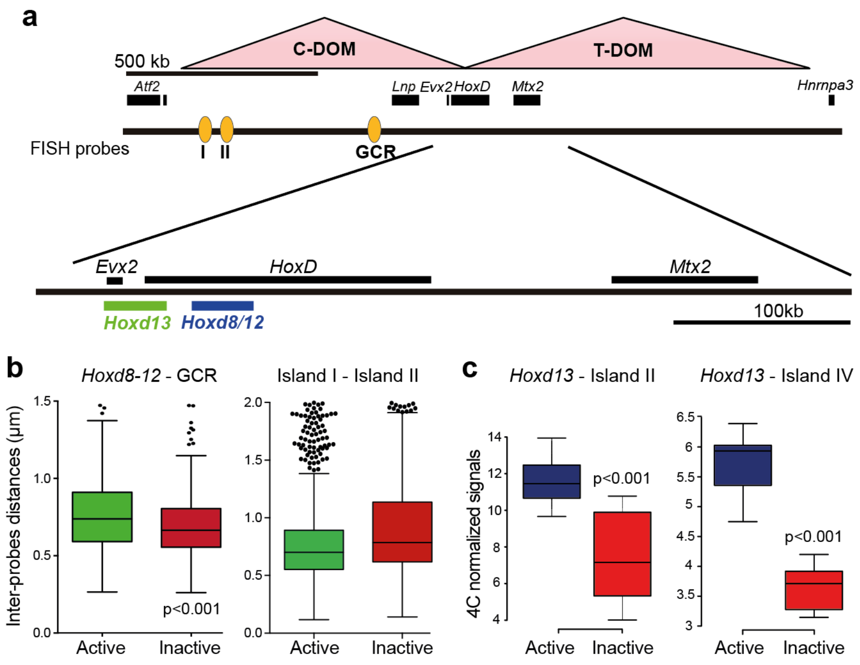

Figure S2: **Quantifications of *Hoxd13* interactions with the regulatory islands using DNA-FISH and 4C-Seq in autopod (active) and zeugopod (inactive) cells. a**, Schematic showing the position of the probes used for the quantification shown below. **b**, 3D-DNA distance measurements for four pairs of fosmid probes: *Hoxd8-Hoxd12*–GCR and island I – island II (Mann-Whitney test). **c**, Quantifications showing the normalized interaction signals from smoothened 4C-Seq either in autopod (active) or in zeugopod (inactive) tissue (pairwise Wilcoxon Rank Sum Tests, followed by BH corrections for multiple testing).

Supplementary Figure S3

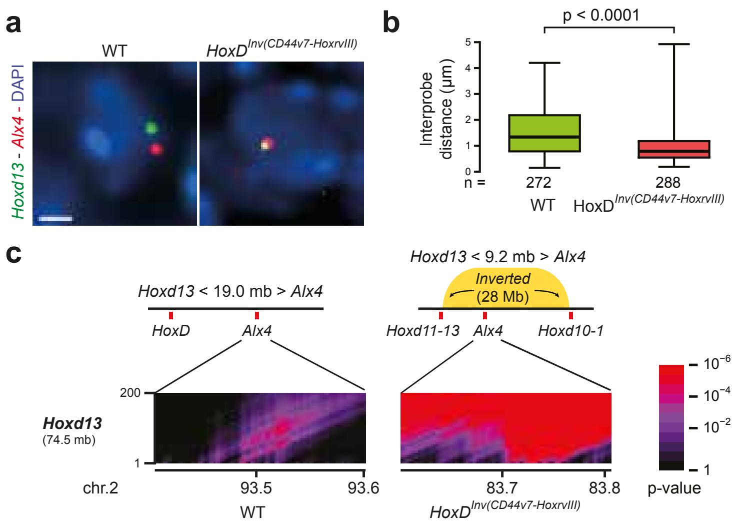

Figure S3: Reducing genomic distances can modulate the contacts between elements located at trans-TAD distances. **a**, DNA-FISH signals showing the greater proximity between *Hoxd13* and *Alx4* in the large telomeric *HoxD*$^{Inv(CD44v7\text{-}HoxrvIII)}$ inversion. **b**, Quantitation of distances as observed in **a** shows a clear increase in proximity in the inverted situation (Mann-Whitney test). **c**, Magnification within a 200 kb large interval centered on *Alx4* on the 4C-seq from *Hoxd13* domainogram showing the increase of contacts in the inverted situation.

Supplementary Figure S4

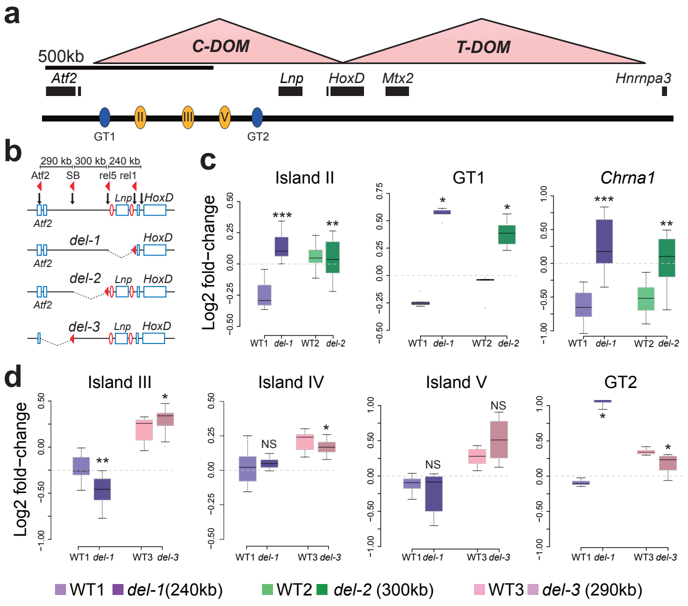

Figure S4: 4C-seq quantitations of *Hoxd13* interactions with its regulatory islands in distal limb cells (autopod). a, Schematic showing the position of the region used for the quantitation of 4C-seq signals shown below. **b**, Schematic showing the breakpoints and the position of the deletions. **c**, Quantitations showing the normalized interaction signals (on three wild type replicates) from 4C-seq for island II, GT1 and *Chrna1* in *del-1* and *del-2* compared to their internal control. **d**, Quantitations showing the normalized interactions signals (on three wild type replicates) from 4C-seq for island III, island V and GT2 in *del-1* and *del-3* compared to their internal control. (* denotes p<0.01 ** denotes p<10-5 and *** denotes p<10-7 compared with Wilcoxon rank sum test).

Supplementary table 1

Gene transcript ID	Mean	WT	Inv(TpSB1-itga6)	FC	p-value	adj. p-value
ENSMUSG00000079277|Hoxd3|1|chr2	78.62	18.11	139.12	7.68	1.10E-07	6.39E-05
ENSMUSG00000027318|Adam33|-1|chr2	37.82	9.06	66.58	7.35	0.0013975	0.12640508
ENSMUSG00000085028|Gm14462|1|chr2	130.92	40.25	221.6	5.5	0.0016833	0.143947728
ENSMUSG00000026950|Neb|-1|chr2	148.86	47.3	250.41	5.29	0.0121658	0.547041645
ENSMUSG00000051747|Ttn|-1|chr2	338.64	123.78	553.49	4.47	0.7025719	1
ENSMUSG00000069049|Eif2s3y|1|chrY	369.02	135.86	602.18	4.43	0.0820751	1
ENSMUSG00000090086|AI480526|-1|chr5	501.5	186.17	816.82	4.39	8.53E-06	0.002573138
ENSMUSG00000021268|Meg3|1|chr12	999.06	378.38	1619.73	4.28	0.0005088	0.061994357
ENSMUSG00000042464|Hoxd4|1|chr2	42.33	16.1	68.57	4.26	0.0001021	0.019848229
**ENSMUSG00000041911|Dlx1|1|chr2**	**533.98**	**215.36**	**852.6**	**3.96**	**0.6070721**	**1**
ENSMUSG00000086878|Miat|-1|chr5	401.98	162.02	641.93	3.96	0.2848759	1
ENSMUSG00000047686|Zcchc5|-1|chrX	281.45	115.73	447.17	3.86	0.5959724	1
ENSMUSG00000047909|Ankrd16|1|chr2	512.6	213.34	811.85	3.81	0.0001608	0.025578472
ENSMUSG00000089726|Mir17hg|1|chr14	242.6	101.64	383.57	3.77	0.7273328	1
ENSMUSG00000020908|Myh3|1|chr11	451.34	190.2	712.48	3.75	0.0865705	1
ENSMUSG00000080316|Ncrna00085|1|chr17	337.77	143.91	531.63	3.69	9.02E-15	3.54E-11
ENSMUSG00000068457|Uty|-1|chrY	326.82	140.89	512.75	3.64	3.28E-11	4.54E-08
ENSMUSG00000059022|Kcp|-1|chr6	523.15	232.46	813.84	3.5	5.19E-139	1.22E-134
**ENSMUSG00000023391|Dlx2|-1|chr2**	**476.86**	**219.38**	**734.34**	**3.35**	**4.03E-23**	**4.74E-19**
ENSMUSG00000056673|Kdm5d|1|chrY	589.49	272.72	906.26	3.32	2.47E-16	1.45E-12
ENSMUSG00000038390|Gpr162|-1|chr6	333.39	158	508.77	3.22	5.56E-16	2.62E-12
ENSMUSG00000015377|Fam116b|-1|chr15	269.61	129.82	409.4	3.15	9.02E-15	3.54E-11
ENSMUSG00000020875|Hoxb9|1|chr11	70.27	34.22	106.33	3.11	9.34E-14	3.14E-10
ENSMUSG00000053332|Gas5|1|chr1	1255.99	625.94	1886.04	3.01	2.13E-13	6.27E-10
ENSMUSG00000090867|Snhg1|1|chr19	588.13	294.86	881.41	2.99	8.37E-13	2.19E-09
ENSMUSG00000034771|Tle2|1|chr10	365.37	187.18	543.55	2.9	1.60E-12	3.14E-09
ENSMUSG00000069045|Ddx3y|-1|chrY	534.41	283.79	785.02	2.77	1.52E-12	3.14E-09
ENSMUSG00000030409|Dmpk|1|chr7	385.35	204.29	566.41	2.77	1.55E-12	3.14E-09
ENSMUSG00000033453|Adamts15|-1|chr9	849.48	453.86	1245.11	2.74	1.75E-12	3.16E-09
ENSMUSG00000085385|9430008C03Rik|-1|chr	537.93	290.83	785.02	2.7	1.08E-11	1.81E-08
ENSMUSG00000055254|Ntrk2|1|chr13	392.85	212.34	573.37	2.7	1.68E-11	2.63E-08
ENSMUSG00000059540|Tcea2|1|chr2	210.92	119.75	302.09	2.52	3.00E-11	4.41E-08
ENSMUSG00000031167|Rbm3|-1|chrX	538.53	307.94	769.12	2.5	3.28E-11	4.54E-08
ENSMUSG00000035967|Ddx26b|1|chrX	684.16	394.48	973.83	2.47	8.98E-11	1.17E-07
ENSMUSG00000006576|Slc4a3|1|chr1	589.44	344.17	834.71	2.43	1.64E-10	2.03E-07
ENSMUSG00000062647|Rpl7a|1|chr2	241.87	142.9	340.84	2.39	2.48E-10	2.91E-07
ENSMUSG00000022822|Abcc5|-1|chr16	1108.69	668.21	1549.18	2.32	3.44E-10	3.86E-07
ENSMUSG00000091793|Rian|1|chr12	590.5	355.24	825.77	2.32	6.07E-10	6.49E-07
ENSMUSG00000024299|Adamts10|1|chr17	1348.12	817.15	1879.09	2.3	9.85E-10	1.01E-06
ENSMUSG00000028248|Sfrs18|1|chr4	1812.61	1123.07	2502.14	2.23	1.05E-09	1.03E-06
ENSMUSG00000041921|Metap1d|1|chr2	327.74	208.31	447.17	2.15	1.97E-09	1.85E-06
ENSMUSG00000027102|Hoxd8|1|chr2	210.52	134.85	286.19	2.12	4.47E-09	4.04E-06
ENSMUSG00000043219|Hoxa6|-1|chr6	166.62	107.68	225.57	2.09	7.92E-09	6.90E-06
ENSMUSG00000092341|Malat1|-1|chr19	2104.82	1370.63	2839	2.07	9.23E-09	7.76E-06
ENSMUSG00000037266|D4Wsu53e|1|chr4	1349	878.53	1819.47	2.07	1.01E-08	8.23E-06
ENSMUSG00000029068|Ccnl2|1|chr4	1135.04	749.72	1520.36	2.03	1.27E-08	9.98E-06
ENSMUSG00000048126|Col6a3|-1|chr1	1595.15	1070.74	2119.56	1.98	1.43E-08	1.08E-05
ENSMUSG00000066842|Hmcn1|-1|chr1	2054.9	1412.9	2696.9	1.91	1.53E-08	1.13E-05

Table S1. Genes up-regulated in the RNA-seq from wild-type *versus* *HoxD*$^{Inv(TpSB1-Itga6)}$ autopods.

Supplementary table 2

Position	Clone ID	Chr	Size (kb)	Start (mm10)	End (mm10)
Dlx1	WI1-562-O4	2	39228	71506627	71545855
Atf2	WI1-1164F22	2	39368	73809214	73848582
Island I	WI1-2854P13	2	37831	73967847	74005678
Island II	WI1-109P4	2	39879	74064904	74104783
Island III	WI1-1404J11	2	40776	74202498	74243274
Island IV-V	WI1-2334J2	2	36664	74259804	74296468
GCR	WI1-2157A11	2	39429	74404558	74443987
Hoxd13	WI1-469P2	2	38845	74636100	74674945
Hoxd8-12	WI1-2777G14	2	38891	74673550	74712441
Alx4	CH29-163J23	2	199950	93468017	93667967

Table S2. List of fosmid and BAC clones used for 3D DNA-FISH. The genomic coordinates are given for mm10.
